# Artificial-Neural-Network-Driven Innovations in Time-Varying Process Diagnosis of Low-K Oxide Deposition

**DOI:** 10.3390/s23198226

**Published:** 2023-10-02

**Authors:** Seunghwan Lee, Yonggyun Park, Pengzhan Liu, Muyoung Kim, Hyeong-U Kim, Taesung Kim

**Affiliations:** 1 School of Mechanical Engineering, Sungkyunkwan University, Suwon 16419, Republic of Korea; hwann96@g.skku.edu (S.L.); ygpark4392@skku.edu (Y.P.); pengzhan@skku.edu (P.L.); 2 Department of Plasma Engineering, Korea Institute of Machinery and Materials (KIMM), Daejeon 34103, Republic of Korea; mkim@kimm.re.kr; 3 SKKU Advanced Institute of Nanotechnology (SAINT), Sungkyunkwan University, Suwon 16419, Republic of Korea

**Keywords:** artificial neural network (ANN), low-k oxide (SiOF), harmonics, high-density plasma (HDP), time varying

## Abstract

To address the challenges in real-time process diagnosis within the semiconductor manufacturing industry, this paper presents a novel machine learning approach for analyzing the time-varying 10th harmonics during the deposition of low-k oxide (SiOF) on a 600 Å undoped silicate glass thin liner using a high-density plasma chemical vapor deposition system. The 10th harmonics, which are high-frequency components 10 times the fundamental frequency, are generated in the plasma sheath because of their nonlinear nature. An artificial neural network with a three-hidden-layer architecture was applied and optimized using k-fold cross-validation to analyze the harmonics generated in the plasma sheath during the deposition process. The model exhibited a binary cross-entropy loss of 0.1277 and achieved an accuracy of 0.9461. This approach enables the accurate prediction of process performance, resulting in significant cost reduction and enhancement of semiconductor manufacturing processes. This model has the potential to improve defect control and yield, thereby benefiting the semiconductor industry. Despite the limitations imposed by the limited dataset, the model demonstrated promising results, and further performance improvements are anticipated with the inclusion of additional data in future studies.

## 1. Introduction

The continuous increase in semiconductor integration has resulted in the increased sensitivity of plasma and inter-reactions owing to its complex surface microscopic topography. The challenges posed by abnormal phenomena in plasma reactors resulting from electrical, chemical, optical, and physical interactions make it difficult to determine the exact cause of this increase. Consequently, defect control and yield improvement are being researched in each process [1,2,3,4,5,6].

Input parameters such as the pressure, gas, power, process gap, and impedance of a process system have been utilized for anomaly detection and equipment diagnosis through trends and statistical management using fault detection and classification (FDC) systems. However, the increasing complexity and number of steps in the process owing to miniaturization have resulted in unexpected results and new variables. In addition, the reliability of process management using sampling measurement data, which were previously used for defect and yield management from wafer to wafer or lot to lot, has decreased. Therefore, real-time process performance diagnosis using data generated from sensors in the system during execution has become necessary.

Real-time process diagnosis has been developed by applying machine learning (ML) techniques using input parameters and process results combined with virtual metrology (VM) technology [7,8,9,10,11,12,13,14]. Recently, plasma-information-based virtual metrology (PI-VM) has been actively researched, and highly accurate virtual models that predict process results by linking plasma factors that affect process characteristics have been developed [15,16,17].

Artificial intelligence (AI) mimics biological structures using neuronal inputs as computing devices and assigns weights that indicate synaptic connection strengths [18,19,20,21,22,23]. Learning is achieved by adjusting the weights assigned to each input. The sensor values and plasma information generated during semiconductor processes are generated as time-varying data, and deep learning is applied in a structured data format that includes label values that reflect the process results. A virtual model formed using deep learning can be used for classification and prediction.

Recently, neural-network-based algorithms have become increasingly popular as methods for anomaly detection and equipment diagnosis, replacing traditional FDC systems. These algorithms can be applied to various types of data, such as images and signals, owing to their ability to solve problems using nonlinear approaches. Examples include regression, modeling, clustering, classification, and big data analysis [24,25,26,27].

The objective of this study was to develop a virtual prediction model utilizing time-varying harmonic data, which are plasma parameters, generated during the low-k oxide (SiOF) deposition process in a high-density (HDP) chemical vapor deposition (CVD) system. An artificial neural network (ANN) model was constructed using a large amount of bias radio frequency (RF) 10th (reverse) harmonic time-varying data obtained through a 1x process. The thickness of the thin liner in the low-k deposition process had a significant impact on the yield, and the thin liner and SiOF were continuously deposited. The objective of the VM model was to classify whether the thickness of the thin liner falls within the normal yield range using the time-varying data of harmonics generated during the process.

Building upon the foundations of previous research in PI-VM, it is evident that the approach to PI parameterization has been instrumental in crafting high-precision VM. This methodology demonstrated significant success in real-world OLED display production [15]. While these contributions have been pivotal in highlighting the potential of PI-VM to overcome challenges commonly observed in statistics-based VM models, our investigation takes a step further. We aim to refine these methodologies and contribute more comprehensively to the existing body of knowledge. With this context in mind, the subsequent sections delve deeper into our unique approach, experiments, and findings.

In this study, we developed the application of an ANN for fault detection through classification. First, we performed labeling based on the results of a process and then created a classification model using the corresponding data. Next, we tested the influence of the hyperparameter values on the model, including the number of hidden layers and nodes in each layer. Finally, we tested various models and determined an optimal classification model using deep learning. With this model, a virtual prediction model with high accuracy can be implemented within 60 s of data collection with cost reduction. Furthermore, our research stands out as it pioneers the use of ANN in the realm of semiconductor fault detection. It underscores the profound impact of hyperparameter tuning on the performance of the model and offers a blueprint for achieving high-precision real-time process monitoring in semiconductor manufacturing.

## 2. Materials and Methods

**Figure 1:**
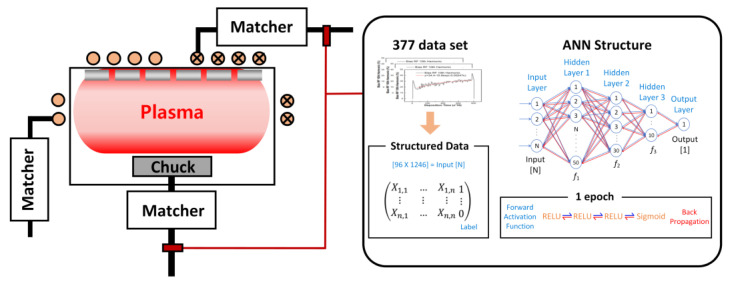
Schematic of the HDP CVD system, illustrating real-time diagnosis using harmonics and an ANN-based model.

SiOF deposition is applied in semiconductor processes to fill gaps between metal lines and prevent the crosstalk of signals in multilayer metal lines. The deposition equipment used in this experiment was an Applied Materials HDP CVD 200 mm (Ultima) system. Figure 1 shows a schematic illustration of the harmonic diagnostic system, demonstrating the combination of harmonics generated during the deposition process and an ANN-based model for real-time process diagnosis. The schematic shows how the time-varying data from the harmonics are input into the ANN model, which then classifies whether the thickness of the thin liner falls within the normal yield range. This integrated approach highlights the use of ANN to detect and diagnose process anomalies based on plasma data, thereby providing a comprehensive view of the proposed diagnostic system. The HDP CVD system is highly productive and capable of both deposition and physical sputtering, which removes overhangs that form on a structure during oxide deposition, enabling bottom-up filling and improving the gap-filling characteristics. Deposition occurs in the intermetal dielectric structure in which low-k materials are applied in the order of silicon-rich oxide (SRO), undoped silicate glass (USG), and SiOF after dry etching of the metal lines.

The SRO and USG thin liners are used to minimize damage from electron-charging plasma-induced damage (PID), and the thickness of the USG thin liner is critical for the yield. While conventional CVD processes use a susceptor (heater) to precisely control the substrate temperature, the HDP CVD system using plasma heating with low-frequency (LF) power has a lower temperature precision than the susceptor method. Therefore, achieving a consistent USG thin-liner thickness is difficult, and the plasma heating step must be strictly controlled for precise temperature management.

In this experiment, the thicknesses of the applied USG thin liner and SiOF material were 600 and 4000 Å, respectively. The deposition temperature was 400 °C, and the process pressure was 5.5 mTorr. Harmonics refer to high frequencies that are integer multiples of the fundamental frequency. The harmonics generated in plasma processes are due to the nonlinear nature of plasma. In this experiment, the harmonics generated in the sheath were formed by applying a bias RF signal to the bottom electrode and were diagnosed. The sheath functioned as a nonlinear capacitor, and the nonlinear characteristics were formed by time-varying changes in the sheath thickness due to plasma oscillations and time-varying changes in the RF field [3,28,29,30]. Harmonics were diagnosed using a directional coupler for nonintrusive measurements. A directional coupler was installed in the transmission line between the RF generator and matcher. The signal picked up by the directional coupler was transmitted to a computer for data processing and transformed into the frequency domain using fast Fourier transform using the ParaDias system (Comdelkorea, Inc., Yongin, Republic of Korea). To diagnose the time-varying behavior, we converted the first fundamental frequency to the second one by dividing it by the Nth frequency power, as shown in Equation (1), and data processing was performed every 0.1 s.

(1) $$\begin{array}{c} N_{th}\;\mathrm{harmonic}\;\mathrm{power}\;\left(\%\right)=\frac{N^{th}frequency\;power}{1^{st}fundamental\;frequency\;power}\times 100 \end{array}$$

In this experiment, the 10th (reverse) harmonic generated during the SiOF oxide deposition process on a 600 Å USG thin liner was diagnosed, and the time-varying behavior of the 10th harmonic was analyzed.

Harmonics are closely associated with the capacitance characteristics of a film formed on the surface or walls of a wafer. When the film surface undergoes electron transfer in the sheath, its increase or decrease is directly proportional to the electron density. Harmonics are generated by the dynamic behavior of electrons in the sheath, and their increase or decrease is directly proportional to the electron density. As the deposition time and thickness of the SiOF film increase, the capacitance of the capacitor decreases exponentially, resulting in a decrease in the charge on the surface. Consequently, the electron density in the sheath increases, resulting in an exponential increase in the 10th harmonic. When the film is thin or absent, it lacks capacitance, resulting in a flat graph without the transient behavior caused by electron charging in the harmonics. Plasma information refers to plasma parameters that are directly correlated with process outcomes. Thin-liner thickness, as a type of plasma information, has a significant impact on the yield and harmonic behavior [4,31]. To evaluate the appropriateness of the thickness of the thin liner, we developed a virtual measurement model using harmonic time-varying data. Generalization is essential to obtain results similar to the training data when applying actual data to a virtual measurement model. In this study, the data corresponding to the maximum and minimum of the 377 harmonic time-varying data points were labeled as normal and included in the training process.

Selecting an appropriate neuron model is crucial for accepting time-varying harmonic data as the input. The selection of a model depends on the nature of the input data. Among the various models, an ANN, which is primarily used for various input data, including images, audio, and raw data, was applied in this study. We adopted the multilayer perceptron (MLP) as the choice of ANN architecture. The decision to use MLP was based on its proficiency in managing structured tabular data, which constituted the majority of our dataset. Moreover, the specific nature of our research task did not demand sequence or image recognition capabilities, making MLP a fitting choice over other architectures such as recurrent neural networks or convolutional neural networks. We believe that the MLP model enabled us to efficiently address the objectives of our study given the dataset characteristics.

The key feature of an ANN model is its ability to make nonlinear decisions. By constructing the model nonlinearly, data that cannot be accessed or analyzed linearly can be accurately predicted. Moreover, although not specifically considered during model construction, an ANN is resilient to noisy data and can be used to identify important features, even in noisy scenarios. For example, when images are used as input data, an ANN model can effectively handle noise and identify features. Moreover, the ANN can adapt to changing input data and learn to recognize new patterns, which is another major advantage. Considering its ability to make nonlinear decisions, robustness against noise, and flexibility in handling different types of inputs, we propose an ANN model.

The presented data consisted of two cases, each containing 600 data points over time. This process involved receiving, converting, and transmitting an input signal. When constructing the ANN, each neuron incorporated a nonlinear function, and the functions connected to each neuron were individually performed using gradient descent methods. Among the various activation functions available for the ANN techniques, those necessary for diagnosing PECVD were selected. In this study, a sigmoid and rectified linear unit (ReLU) were utilized for the hidden layers to ensure high-quality training of the model.

The sigmoid activation function is nonlinear and characterized by a gentle curve compared with the step function. Unlike step functions that exhibit dramatic output changes based on boundaries, the sigmoid function changes smoothly, which is vital in neural network learning; this was the reason for using the sigmoid function for activation.

The sigmoid function is mathematically expressed as

(2) $$\begin{array}{c} h\left(x\right)=\frac{1}{1\;+\;\exp\left(-x\right)} \end{array}$$

**Figure 2:**
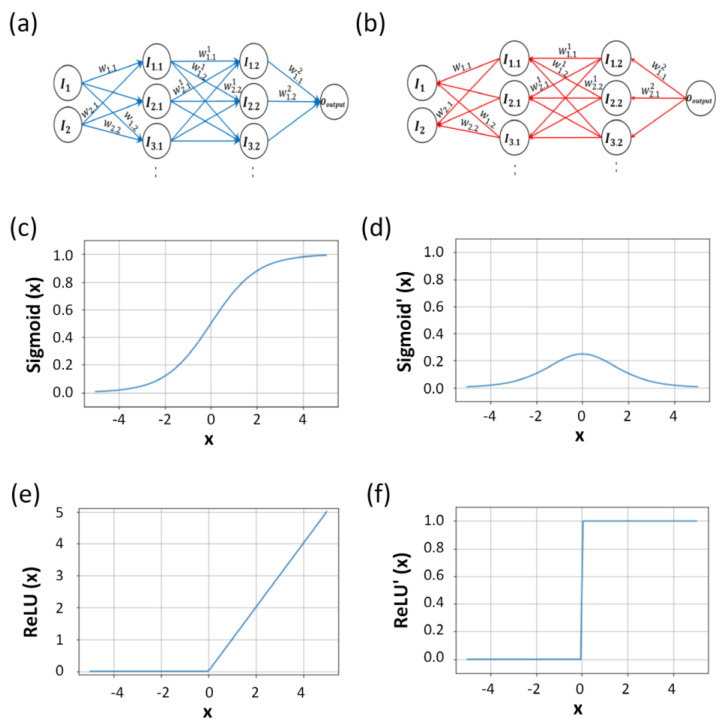
Processes contributing to the progression of an epoch: (**a**) forward propagation, (**b**) backward propagation and associated activation functions, (**c**) sigmoid activation function, (**d**) derivative of sigmoid, (**e**) ReLU activation function, and (**f**) derivative of ReLU.

The sigmoid function ranges between 0 and 1, preventing the occurrence of extreme values. Unlike other activation functions, the intermediate value of the sigmoid function is 0.5, as shown in Figure 2c. This intermediate value of 0.5 is advantageous for binary classification. Additionally, the sigmoid function can differentiate between two classes, with values close to 1 and 0. The 0.5 intermediate value of the sigmoid function serves as the threshold for classifying a model with high accuracy [32]. As the research progressed, various activation functions were developed. The deeper the layer of the model, the weaker the gradient. The gradient vanishing problem must be addressed, because the error rate is difficult to calculate at a large depth of the hidden layer. The mathematical expression of the ReLU function is

(3) $$\begin{array}{c} y=\left(\begin{array}{cc} x, & \left(x>0\right)\\ 0, & \left(x\leq 0\right) \end{array}\right)\; \end{array}$$

Figure 2e shows the ReLU, which is commonly used as an activation function for the hidden layers of neural networks [33]. At the end of the third-order hidden-layer ANN model, the weight parameters were used for the sigmoid classification. When the output layer has a sigmoid activation function, the role of the activation function used in the hidden layer is to update the weight for forward propagation, which is achieved using the ReLU. The process from the input parameter to the final predicted value is described as propagation, as shown in Figure 2a, where the input value has a weight across all layers, including the hidden and output layers. The input passes through the layers and gains additional weight. Finally, it passes through the last output layer, the sigmoid activation function, and transforms into the final value. The value of each node in the forward propagation activation function is expressed as

(4) $$\begin{array}{c} I_{1.1}=ReLU{(I}_{1}w_{1.1}+I_{2}w_{2.1})\; \end{array}$$

where $I_{1.1}$ is the input value through the activation function, $I_{1}$ is input parameter 1, $I_{2}$ is input parameter 2, $w_{1.1}$ is the weight of input parameter 1, and $w_{2.1}$ is the weight of input parameter 2.

(5) $$\begin{array}{c} I_{2.1}={ReLU{(\;I}_{1}(I}_{1}\;w_{1.2}+I_{2}\;w_{2.2})\; \end{array}$$

where $I_{2.1}$ is the input value through the activation function, $I_{1}$ is input parameter 1, $I_{2}$ is input parameter 2, $w_{1.2}$ is the second-order weight of input parameter 1, and $w_{2.2}$ is the second-order weight of input parameter 2.

(6) $$\begin{array}{c} I_{1.2}={ReLU(I}_{1.1}w_{1.1}^{1}+I_{2.1}w_{2.1}^{1}+\cdots ) \end{array}$$

where $I_{1.2}$ is the input value through the second hidden-layer activation function, $w_{1.1}^{1}$ is the weight of $I_{1.1}$, and $w_{2.1}^{1}$ is the weight of $I_{2.1}$. The processes of I, the nodes, and the weights occur sequentially. The equation associated with the sigmoid function and weight in the final output layer is

(7) $$\begin{array}{c} O_{output}=Sigmoid{(I}_{1.2}w_{1.2}^{2}+I_{2.2}w_{2.2\;}^{2}+\cdots ) \end{array}$$

where $O_{output}$ is the predicted value, $I_{2.2}$ is the input value through the second hidden-layer activation function, $w_{1.2}^{2}$ is the weight of $I_{1.2}$ and $w_{2.2}^{2}$ is the weight of $I_{2.2.}$ The cost function is equal to the mean of the square of the difference between the predicted and actual values. This function is the criterion for determining model accuracy and is expressed as

(8) $$\begin{array}{c} R_{c}=\sum \frac{{{(O}_{output}-V_{real})}^{2}}{2}\; \end{array}$$

Here, $R_{c}$ is the cost function value, and $V_{real}$ is the actual value.

The value derived from the input value to the output value is considered a single forward propagation. After the forward propagation, the predicted value can be compared with the actual value obtained using the loss function. Thus, we obtain the cost function by adding the loss for each value, which is expressed as $R_{c}$. This is shown in Equation (8) and is the output result of forward propagation. Table 1 lists the model results when only forward propagation was performed. When forward propagation was directed from the input layer to the output layer, backpropagation was calculated in the opposite direction from the output layer to the input layer to update the weight, as shown in Figure 2b [34].

The derivative of the sigmoid function shown in Figure 2d for backward propagation is

(9) $$\begin{array}{c} {h\left(x\right)}^{\prime }=h\left(x\right)\left(1-h\left(x\right)\right)\; \end{array}$$

The sigmoid function is expressed as an exponential function. The response ranges from 0 to 1, and the derivative of the sigmoid function ranges from 0 to 0.25. In the graph of the derivative of the sigmoid function, the maximum is 0.25, and the minimum converges to 0. The differential of the sigmoid function is multiplied in series from backpropagation to the layer in front of the input layer. When the value is less than 1, the multiple gradually decreases [35,36]. As the depth of the network increases, this can lead to rapid diminishing of gradients, a phenomenon termed the vanishing gradient problem. This poses challenges in training deeper neural networks when the sigmoid function is employed.

The derivative of the ReLU for backward propagation is

(10) $$\begin{array}{c} \frac{\partial y}{\partial x}=\left(\begin{array}{cc} 1, & \left(x>0\right)\\ 0, & \left(x\leq 0\right) \end{array}\right)\; \end{array}$$

The ReLU activation function is used to reduce gradient vanishing problems for the hidden layer. It also has the advantage of not decreasing the slope when outputting 1, because the derivative of the ReLU function has an output of either 0 or 1, as shown in Figure 2f.

The derivative for the ReLU activation function is shown in Equation (10). This derivative outputs either 0 or 1 depending on its input, ensuring that the gradient does not vanish rapidly even in deep networks. But there is a catch when the input is negative, the gradient is 0, introducing a potential problem where certain neurons might become inactive or “die” during training. While several variants of the ReLU have been proposed to address this issue, the simplicity and efficiency of the basic ReLU function make it a popular choice in many neural network architectures.

Both derivatives of these activation functions encapsulate the characteristics of their respective functions and provide the necessary gradient for updating the network’s weights. Understanding the strengths and weaknesses of each allows for their optimal application, ensuring efficient training of neural networks.

The weight at backpropagation from the cost function is calculated as

(11) $$\begin{array}{c} \frac{{\partial R}_{c}}{\partial O_{output}}=\frac{{\partial R}_{1}}{\partial O_{output}}=\frac{1}{2}\frac{\partial }{\partial O_{output}}{{(O}_{output}-V_{real})}^{2}={(O}_{output}-V_{real})\\ \frac{\partial O_{output}}{{\partial (I}_{1.2}w_{1.1}^{2}+I_{2.2}w_{2.1}^{2})}=h(I_{1.2}w_{1.1}^{2}+I_{2.2}w_{2.1}^{2})(1-h(I_{1.2}w_{1.1}^{2}+I_{2.2}w_{2.1}^{2}))=O_{output}(1-O_{output})\\ \frac{{\partial I}_{1.2}w_{1.1}^{2}+I_{2.2}w_{2.1}^{2}}{\partial w_{1.1}^{2}}=I_{1.2}\\ \frac{{\partial R}_{1}}{\partial O_{output}}={(O}_{output}-V_{real})\;\times \;O_{output}(1-O_{output})\;\times \;I_{1.2}\\ \emptyset_{1}^{3}=\frac{{\partial R}_{1}}{{\partial I}_{1.2}}={(O}_{output}-V_{real})\;\times \;O_{output}(1-O_{output})\\ \;\;\;\;\;\;\;\;\;\;\;\;\;\;\;\;\;\;\;\;\;\;\;\;\;\;\;\;\;\;\;\;\;\;\;\;\;\;\;\;\;\;\;\;\;\;\;\;\;\;\;\;\;\;\;\;w_{1.1}^{2}=w_{1.1}^{2}-\emptyset_{1}^{3}{\times I}_{1.2}\; \end{array}$$

where $\emptyset_{1}^{3}$ is the derivative of $R_{c}$ divided by the derivative of ${\partial I}_{1.2}$. The weight value is updated in reverse as the quotient of the derivative value of the activation function to that of the cost function. This is the first weight updating method for backpropagation.

Equation (11) describes the weight updating method from the cost function to the third hidden layer. When the weight update passes through the derivatives of the sigmoid and ReLU and arrives at backpropagation, one epoch is said to have been completed. The weight update value obtained in the process is applied from the input parameter layer to the forward- and back-propagation processes. The calculation of the cost function using the predicted value obtained by forward propagation conducted with a weight update and backpropagation represents two epochs. Thus, an epoch involves updating the weight value to optimize the cost function value, which is the difference between the predicted and actual values.

After completing the model construction, it was verified using independent data. Although it is preferable to include a large amount of data in model construction, the use of the data for learning can result in overfitting. Therefore, further verification is required [14].

## 3. Results

**Figure 3:**
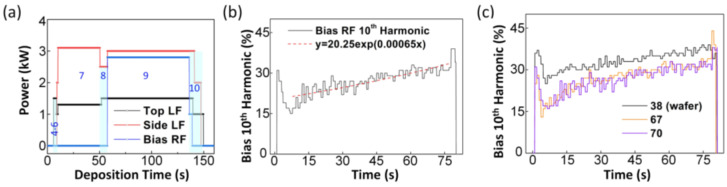
(**a**) Sequence of low-frequency (LF) and radio-frequency (RF) power, (**b**) 10th bias RF harmonics, and (**c**) classification according to binary classification; 38 wafer represents Case 1, and 67 and 70 wafers represent Case 2.

An LF of 2 MHz was used in the deposition process for the top and side coils at each stage, as shown in Figure 3a. Table 2 presents the sequence of the SiOF oxide deposition. To understand the variation in harmonic intensity in response to time-varying factors, we examined the temporal patterns of the data flow. Figure 3b shows that the 10th harmonic RF bias at 135.6 MHz exhibited an exponential increase. Owing to the limited sample size of the 377 wafer data points available for testing, the accuracy and loss values of the ANN model could be significantly influenced by the data used for training. Therefore, fitting the model to the input data rather than adjusting the data to fit the model is crucial. The choice of data has a greater impact on the model than the model architecture itself.

**Figure 4:**
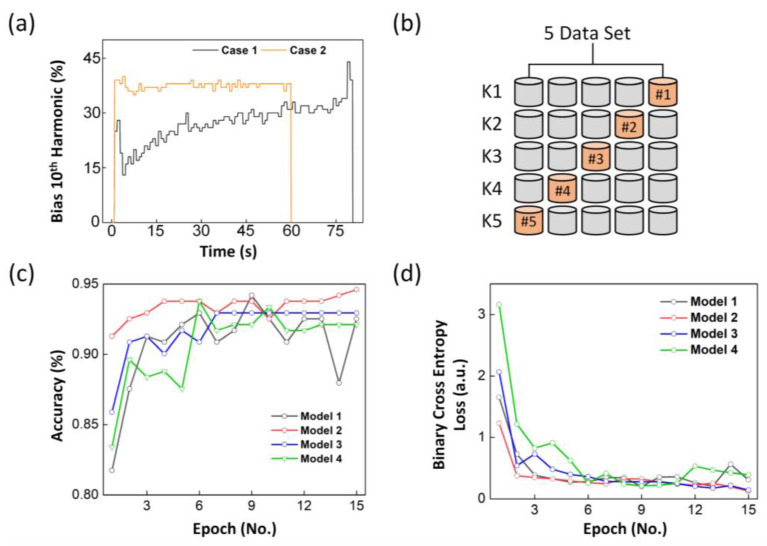
(**a**) Representative of binary classification cases, (**b**) schematic of K-fold cross-validation process, epoch-driven results, (**c**) accuracy, and (**d**) BCEL.

This approach was employed to prevent problems that may result from modeling with limited data. In summary, to ensure the accuracy of an ANN model, it is imperative to carefully select the appropriate input data rather than focus on the model architecture. Figure 3c depicts two binary cases, each exhibiting a distinct graphical pattern. Additionally, Figure 4a also shows two binary cases. The K-fold cross-validation method can be employed to address the limitations associated with the limited data [37,38,39]. The value of K used for model verification is not fixed, and values ranging from 5 to 10 are commonly used [38]. For the model used here, the value of K was set to 5. The main reason for using K-fold cross-validation, as shown in Figure 4b, is to improve the accuracy for datasets with a smaller number of data points. By using all the data for verification instead of dividing them into training, validation, and test sets, a model with improved performance can be learned. Through repeated verification calculations, the optimal model can be identified based on the error values recorded during each cycle. Validation also plays a role in preventing overfitting, which occurs when a model fits well with a specific training set but performs poorly with a test set [40]. The disadvantage of K-fold cross-validation is that it requires more time than the general learning method performed with general training and test sets.

After model verification, quantitative figures were obtained to assess the model fit. Various values such as the mean squared error, R^2^ score, mean absolute error, and binary cross-entropy loss (BCEL) were calculated to determine the fit. The BCEL method was used to determine the fit. The BCEL method was used to evaluate the fit of the current model. The BCEL function is commonly used for binary classification problems [41,42]. Unlike the R^2^ score, which is a commonly used evaluation metric for regression models, the BCEL function measures the error between the predicted probability and actual label, indicating the accuracy of the model predictions. The BCEL value ranges from 0 to 1, with a higher value indicating a more accurate prediction. To validate the model in this study, we used harmonic data from Harmonics Inc. as the input for binary classification, and the BCEL function was employed, as shown in Equation (12).

(12) $$\begin{array}{c} \mathrm{BCEL}\;=\;-\frac{1}{N}\overset{N}{\underset{i=1}{\sum }}y_{i}\;*\;log\hat{y_{i}}\;+\;\left(1-y_{i}\right)\;*\;\log\;(1-\hat{y_{i}}) \end{array}$$

where $y_{i}$ is a real value and $\hat{y_{i}}$ is the estimated value from the harmonic VM model. Increasing the number of epochs decreased the BCEL value.

Figure 4c,d shows the values obtained through K-fold cross-validation and shows that increasing the number of epochs gradually decreased the cost function through weight updating based on forward propagation and backpropagation. The result indicates good model suitability based on BCEL. The loss function represents the difference between a model’s probability distribution and the actual probability distribution of the data. Therefore, the closer this value is to zero, the higher the accuracy of the model. Figure 4 confirms that no significant change occurred in accuracy after 15 epochs. More epochs may slightly increase the accuracy values, but they can lead to overfitting and adversely affect the results. The same concept applies to the loss function, where the loss value may decrease but can indicate overfitting. Table 3 provides detailed results of the model over 15 epochs, determining the best model as that of epoch 15 and identifying it as the final harmonic VM model.

## 4. Conclusions

This paper presents a novel approach for predicting SiOF deposition in PECVD chambers, emphasizing the significance of SiOF materials in the semiconductor industry. A Python-based VM model utilizing an ANN was developed by incorporating preprocessing techniques to analyze the time-varying data. The ANN model employed a sigmoid activation function for the output layer and a ReLU function for the hidden layers. The model was validated using K-fold cross-validation. The final VM model exhibited impressive performance, achieving a BCEL value of 0.1277 and an accuracy of 0.9461. These results demonstrate the potential of ANN-based VM models for an effective process diagnosis in SiOF deposition. By enhancing data quality and reliability, this study contributes to ongoing advancements in process diagnosis techniques within the semiconductor industry. The developed VM model creates new avenues for improved manufacturing processes and quality control during SiOF deposition. It serves as a valuable tool for optimizing production efficiency and ensuring high-quality semiconductor devices. Further research and development in this field will continue to enhance the accuracy and applicability of VM models, resulting in advancements in semiconductor manufacturing and contributing to the overall expansion of the industry.

## Figures and Tables

**Table 1:** Results of first-order forward propagation (without backpropagation).

Start	Model 1 (3 Hidden Layers)	Model 2 (3 Hidden Layers)	Model 3 (3 Hidden Layers)	Model 4 (3 Hidden Layers)
Starting Node	200	100	100	200
Loss	1.6537	1.2331	2.0683	3.1615
Accuracy	0.8174	0.912863	0.8589	0.8340

**Table 2:** Sequence of the deposition process.

No.	Step	Gas	Power
1	Chamber cleaning	SF_6_	Microwave (GHz)
2	Chamber seasoning	SiH_4_, Ar, O_2_	Top & Side LF
3	Wafer loading	-	-
4	Pressure stabilization	Ar, O_2_	-
5	Plasma on	Ar, O_2_	Top and Side LF
6	Throttle valve on	Ar, O_2_	Top LF Ramp-up
7	Preheating for heat-up	Ar, O_2_	Top and Side LF
8	SRO and USG deposition	SiH_4_, Ar, O_2_	Top and Side LF
9	SiOF deposition	SiH_4_, SiF_4_, Ar, O_2_	Top and Side LF, RF
10	Transition	-	Reduced Top and Side LF, RF
11	Power and gas off	-	-

**Table 3:** Results of numbers of layers and nodes (15 epochs).

Epoch No. 15	Model 1 (3 Hidden Layers)	Model 2 (3 Hidden Layers)	Model 3 (3 Hidden Layers)	Model 4 (3 Hidden Layers)
Starting node	200	100	100	200
Loss	0.3076	0.1277	0.1433	0.3922
Accuracy	0.9253	0.9461	0.9295	0.9212

## Data Availability

Not applicable.
